# Colored thermal camouflage and anti-counterfeiting with programmable In_3_SbTe_2_ platform

**DOI:** 10.1515/nanoph-2023-0924

**Published:** 2024-02-28

**Authors:** Sihong Zhou, Shikui Dong, Yanming Guo, Yong Shuai, He-Xiu Xu, Guangwei Hu

**Affiliations:** Key Laboratory of Aerospace Thermophysics of Ministry of Industry and Information Technology, Harbin 150001, China; School of Energy Science and Engineering, 47822 Harbin Institute of Technology, Harbin 150001, China; School of Electrical and Electronic Engineering, 54761 Nanyang Technological University, 50 Nanyang Avenue, Singapore, 639798, Singapore; Air and Missile Defense College, Air Force Engineering University, Xi’an 710051, China

**Keywords:** IST phase change material, industry-friendly platform, resonance mode coupling, VIS-IR compatible camouflage, thermal anti-counterfeiting

## Abstract

Camouflage is an important technology in various scenarios. Usually, this involves the visible compatibility of the background, which however is facile under infrared thermal radiation detection. The simultaneous visible and thermal camouflage are challenging because it requires full and decoupled manipulations of visible reflection and infrared emissivity using one single device, let alone to its adaptivity to complex environments. Here, we report a programmable, colored thermal camouflage at 3–5 μm and 8–14 μm based on mode coupling in phase-change In_3_SbTe_2_ materials. A series of industry-friendly colored multilayer thermal emitters are designed consisting of an anti-reflectance layer for structure coloration above a coupled nanocavity for IR modulation, which easily realizes the complete decoupled control of visible color and infrared emissivity. Our solution features independent structural visible colors in the full visible range and continuously programmable dual-band emissivity modulation with up to 90 % absolute tuning range. Our work facilitates near optimal camouflage and anti-counterfeiting solution for visible-infrared multi-band compatibility of complex environments under different temperatures and colored appearances.

## 1 Introduction

**Figure 1:**
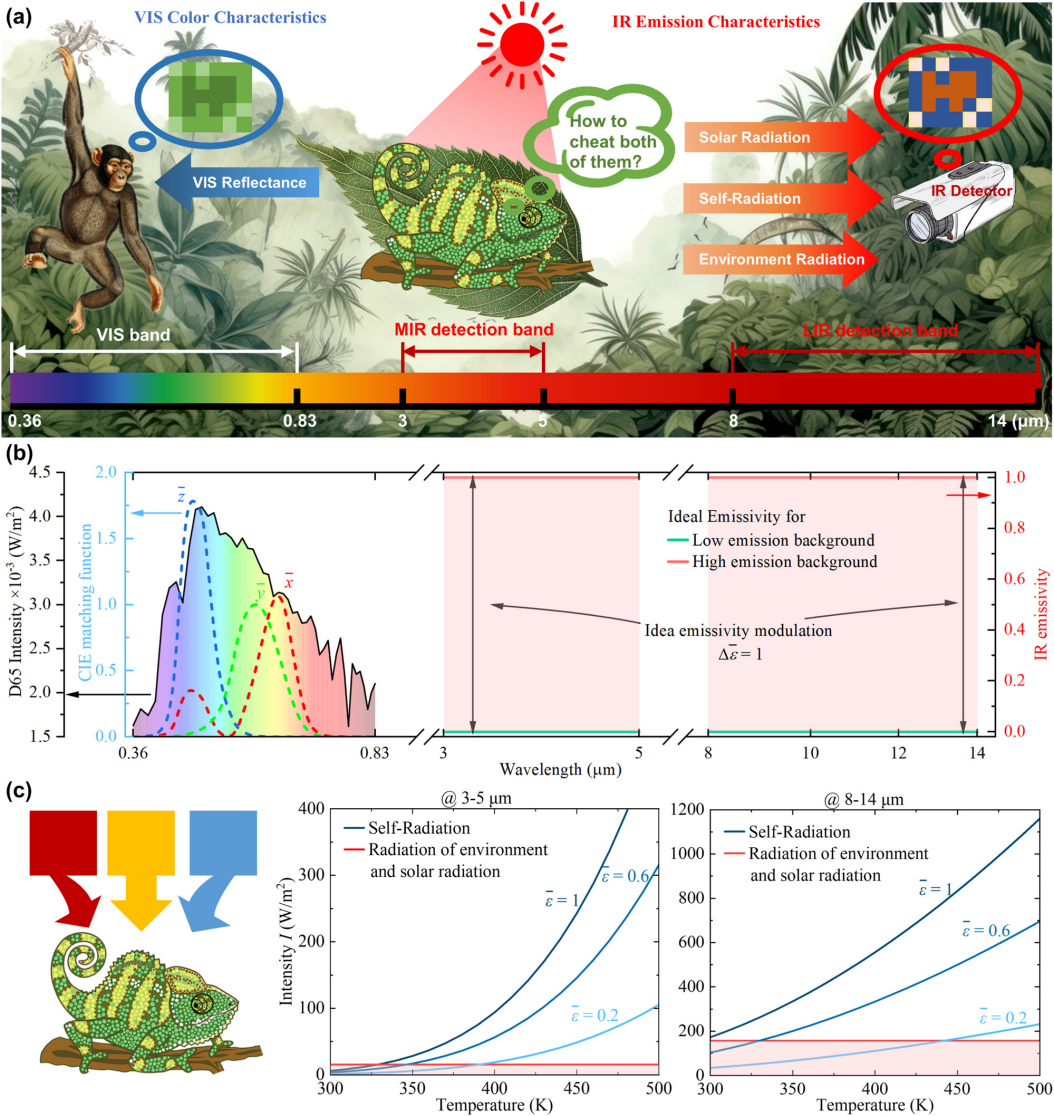
VIS and IR camouflage. (a) Schematic diagram of color camouflage and IR detection. (b) Schematic diagram of D65 light source power density, the dash lines are CIE matching function, and the ideal IR emission modulation ability at 3–5 μm and 8–14 μm. (c) The color camouflage, the band-integrated environment and solar radiance, and the self-radiation of objects with different emissivity under different temperatures.

Due to the difficult compatibility of spectral signatures, the camouflage object targeted at single band is easy to be detected at other bands. In Figure 1(a), adaptive visible (VIS) color camouflage inspired by chameleons [1] can achieve VIS camouflage in the complex visual background, which however can be easily identified in the infrared (IR) detection. The basic solution of the problem is the continuously programmable colored IR compatible camouflage. This would favor not only the ideal VIS color characteristic (0.36–0.83 μm) but also the outstanding IR emission modulation ability at both dual-detection bands (3–5 μm and 8–14 μm), which preferably can be continuously and largely tunable to adapt to the varying complex environment.

Extensive studies have been reported to achieve discrete radiation characteristics [2] by using photonic crystals [3], [4], [5], [6], metal–semiconductor–metal nanocavity [7], [8], [9], porous nanostructures [10], and coding metasurfaces [11]. Among them, multispectral complementary technology is commonly utilized to realize all-band camouflage by integrating the dielectric multilayers or metal nanostructure of different spectral regions [12], [13], [14], [15]. However, this approach is limited by complex geometry, and requires matching of multiple materials to achieve a delicate balance of different camouflage requirements within different bands. So far, continuously programmable colored thermal camouflage remains less explored for several reasons. Firstly, traditional materials are not able to dynamically regulate radiation due to the static electronic state density [15], [16], [17], [18]. Moreover, the interaction between different materials and structures makes it difficult to realize wide-band decoupled control, and thus it requires dedicated trade-off between VIS colors and IR emission modulation ability.

Another important concern to design camouflage is the adaptability to the complex surroundings particularly in infrared windows. In close analog to the VIS mosaic camouflage, the “thermal mosaic camouflage” was proposed by spatially modulating the thermal radiation characteristics of the disguised object to confuse the detector [19], [20], which requires spatial modulation of the thermal radiation of camouflage devices. Impressively, the newest non-volatile plasmonic phase-change materias (PCM) – In_3_SbTe_2_ (IST) – could adaptively modulate whole IR radiation characteristics via manipulating light–matter interactions [21] and realize space programmable radiation by local phase change [22]. It has been adopted to achieve thermal radiation switch [23] and single band thermal camouflage [19]. Compared with common phase-transition materials such as VO_2_ [24], [25], IST does not need continuous energy supplementation to sustain optical characteristics after the phase change. Besides, compared with Ge_2_Sb_2_Te_5_, and Ge_3_Sb_2_Te_6_ [26], [27], [28], amorphous IST (aIST) is a lossless dielectric material, and crystalline IST (cIST) manifests metallic characteristic at the whole IR region due to unconventional metavalent bonding mechanism, affording fundamentals for achieving more widely tunable IR radiation management than conventional PCMs [29], [30]. But cIST is still lossless at VIS spectral region and is a little different from aIST. This unique property makes it possible to decouple the thermal radiation modulation of the VIS-IR multi-spectral regions. By controlling the local phase change range of cIST, it is possible to achieve continuous programmable IR emissivity regulation with little impact on VIS color characteristics.

Here, a multilayer mode coupling thermal emitter (MCTE) is proposed and optimized by a general physics-assisted intelligent optimization method for the colored thermal emission modulator to achieve VIS-IR compatible mosaic thermal camouflage. By adjusting the material composition and thickness of each layer, the VIS reflectance can be modulated to realize almost all color, while near 90 % emissivity regulation is guaranteed in two atmosphere-transparent bands of 3–5 μm and 8–14 μm. Programmable and continuous regulation of emissivity in IR detection band can be achieved by local phase change of IST materials. Most importantly, the VIS light color characteristic of the device is not affected by the IR emission modulation, realizing independent control of VIS-IR region. Our strategy affords a promising method and platform for programmable modulating VIS-IR multiband emission characteristics and offers a reference for design and applications of programmable optical devices.

## 2 Results and discussion

### 2.1 Principle of colored IR programmable camouflage

We start with the VIS color mosaic camouflage. The CIE 1931 standard *xy* chrominance diagram is used to mathematically characterize the color. Their values are [31]:

(1a) $$x=\frac{X}{X+Y+Z}$$

(1b) $$y=\frac{Y}{X+Y+Z}$$

where *X*, *Y*, and *Z* are three stimulus values for three primary colors (red, green, and blue), which can be expressed as

(2a) $$X=100\frac{\underset{\lambda }{\int }S\left(\lambda \right)R\left(\lambda \right)\bar{x}\left(\lambda \right)d\lambda }{\underset{\lambda }{\int }S\left(\lambda \right)\bar{y}\left(\lambda \right)d\lambda }$$

(2b) $$Y=100\frac{\underset{\lambda }{\int }S\left(\lambda \right)R\left(\lambda \right)\bar{y}\left(\lambda \right)d\lambda }{\underset{\lambda }{\int }S\left(\lambda \right)\bar{y}\left(\lambda \right)d\lambda }$$

(2c) $$Z=100\frac{\underset{\lambda }{\int }S\left(\lambda \right)R\left(\lambda \right)\bar{z}\left(\lambda \right)d\lambda }{\underset{\lambda }{\int }S\left(\lambda \right)\bar{y}\left(\lambda \right)d\lambda }$$

where the wavelength range is 0.36–0.83 μm, *S*(*λ*) is the standard spectral radiation energy distribution of the D65 light source, and $\bar{x}$ , $\bar{y}$ , and $\bar{z}$ are CIE matching functions shown in Figure 1(b). By regulating VIS reflectance *R*(*λ*), we can realize active control of VIS color characteristics.

For IR camouflage, we note the raw radiation intensity *I* recorded by the IR detector will be auto-scaled to the maximum (*I*_max_) and minimum (*I*_min_) values of the acquired images. As a result, the normalized radiation intensity *I*_norm_ should be adopted as indicative signals for camouflage applications [20], [32].

(3a) $$\begin{array}{ll} I\left(\varepsilon \left(\lambda \right),T\right) & =\overset{\lambda_{\max}}{\underset{\lambda_{\min}}{\int }}\frac{\varepsilon \left(\lambda \right)c_{1}\lambda^{-5}}{\exp\left(c_{2}\;/\lambda T\right)-1}+\left(1-\varepsilon \left(\lambda \right)\right)\\ & \;\times \left(\frac{\varepsilon_{\text{amb}}\left(\lambda \right)c_{1}\lambda^{-5}}{\exp\left(c_{2}\;/\lambda T_{\text{amb}}\right)-1}+I_{\text{sun}}\right)\text{d}\lambda \end{array}$$

(3b) $$I_{\mathrm{norm}}=\frac{I-I_{\min}}{I_{\max}-I_{\min}}$$

where *ε*(*λ*), *ε*_amb_ (*λ*), *T*, and *T*_amb_ are spectral emissivity and temperature of the object and ambient, respectively. *c*_1_ = 3.7419 × 10^16^ W m^2^ and *c*_2_ = 1.4388 × 10^−2^ m K are the first and second radiation constant [33]. The minimum and maximum wavelength are *λ*_min_ = 3 μm and *λ*_max_ = 5 μm within 3–5 μm band while they are *λ*_min_ = 8 μm and *λ*_max_ = 14 μm within 8–14 μm band. In Eq. (3a), the first term is self-radiation, the second term is total reflectance of direct solar and ambient radiation, and *I*_sun_ is the direct solar radiation intensity calculated by MODTRAN used for analysis of optical measurements through atmosphere [34] whose detailed dates are shown in Supplementary S1.

To “fool” the detector, the self-radiation, and the reflectance of solar and surrounding environment radiation have contributions, which vary as the temperature and emissivity of the camouflaged object. According to Eq. (3a), there is also a competitive relationship between self-radiation and total solar and environmental radiation, which determines who plays the main role. As shown in Figure 1(c), the sum of direct solar and environment radiation is ∼15.64 W/m^2^ at 3–5 μm and ∼156.19 W/m^2^ at 8–14 μm when the ambient temperature is *T*_amb_ = 300 K. For camouflaged objects with high temperature, the predominant source of IR signal is self-radiation, which is positively correlated with the emissivity of the camouflaged object. On the contrary, the total direct solar and environment radiation play a major role at 3–5 μm so that the IR signal is negatively correlated with emissivity of the camouflaged object when its temperature is the same as the background. Since the blackbody emission is still large than total direct solar and ambient radiation at 8–14 μm, the IR signal at 8–14 μm is positively correlated with the emissivity. This opposite trend does not affect our ability to achieve programmable infrared camouflage, because we aim to achieve IR “mosaic” camouflage which does not require the same spectral as the ambient background. Moreover, this interesting phenomenon provides us another interesting capability, i.e., thermal anti-counterfeiting. Parts with the same emissivity will show different infrared signals at 3–5 μm and 8–14 μm, which will be detailed later.

### 2.2 Structure design and optimization

**Figure 2:**
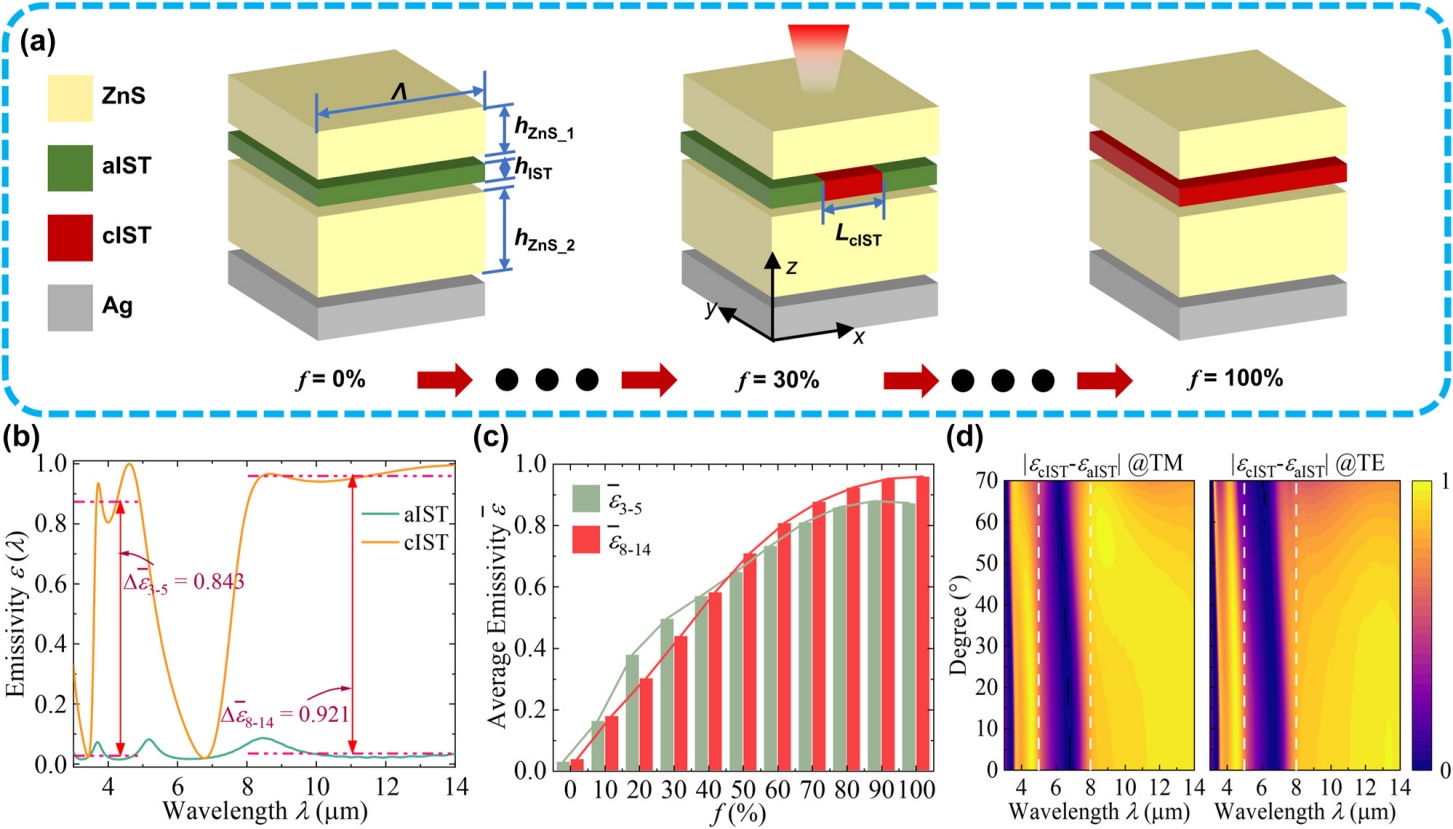
The modulation of IR emission. (a) Structure of four-layer ZnS/IST/ZnS/Ag MCTE and modulation method of local phase change, *L*_cIST_ is the width of cIST region; (b) spectral IR emissivity *ε*(*λ*) before and after phase change, the red dashed lines show the average emissivity $\bar{\varepsilon }$ at 3–5 μm and 8–14 μm. (c) Polarization-averaged average emissivity with different fill factor *f*. (d) Spectral emissivity difference under different polarization and incident angles.

Resonance mode coupling affords a simple and efficient means for achieving special optical characteristics which depends on the superposition of multiple resonance modes rather than on specific complex structures [35], [36], [37]. As shown in Figure 2(a), the simplest resonance mode coupling of thermal emitters can be constructed by inserting IST layer in ZnS layers. Both top ZnS layers and IST/ZnS/Ag nanocavity exhibit their own resonant modes, and mode coupling will produce new optical resonance characteristics. Different from other phase change materials, aIST and cIST exhibit positive and negative real parts of permittivity at 3–14 µm, respectively, which allows for continuous modification of emission difference by varying the composition, particularly the fill factor *f* of the cIST in a structure cell period Λ (*f* = *L*_cIST_/Λ). By replacing the ZnS layer with a multilayer distributed Bragg reflector (DBR), a variety of colors can be achieved while maintaining similar great infrared modulation capability, which will be discussed later.

Since both resonance modes of anti-reflectance (AR) layers and nanocavity are sensitive to the thickness of each layer, we first demonstrate the extraordinary emissivity modulation ranges before and after IST phase change within 3–5 μm and 8–14 μm by optimizing the thickness of the ZnS AR layer *h*_ZnS_1_, the thickness of IST layer *h*_IST_ and middle ZnS layer *h*_ZnS_. We use a general physics-assisted intelligent optimization method, i.e. the GPU transmission matrix method and genetic algorithm (GPU-TMM-GA) method (see more details in Supplementary S2) [19]. Our structures are reciprocal, and the spectral emissivity *ε*(*λ*) is equal to the spectral absorptance *α*(*λ*) [38]. The permittivity of ZnS and Ag are taken from Refs. [39], [40], and the permittivity of IST is obtained from the experimentally fitted Lorentz–Drude model in Ref. [21]. Detailed dates can be referred to Supplementary S3.

To obtain huge IR emission modulation ability, the fitness value *F*_adopt_ can be expressed as:

(4) $$F_{\text{adopt}}=\max\left(0.4\left|\Delta {\bar{\varepsilon }}_{3-5}\right|-0.2\left|\Delta {\bar{\varepsilon }}_{5-8}\right|+0.4\left|\Delta {\bar{\varepsilon }}_{8-14}\right|\right)$$

where $\Delta {\bar{\varepsilon }}_{3-5}$ , $\Delta {\bar{\varepsilon }}_{5-8}$ , and $\Delta {\bar{\varepsilon }}_{8-14}$ are average emissivity difference before and after the phase change at 3–5 μm, 5–8 μm, and 8–14 μm, respectively. Simple additive weighting is exploied here to calculate the fitness value of multi-objective optimization, which focuses on optimizing some objectives by controlling the weight of each optimization objective [41]. To achieve the maximum emissivity modulation ability within 3–5 μm and 8–14 μm, we set the weight before the emissivity difference between the two bands to the same larger value as 0.4. The optimization goal of maintaining the emissivity in the 5–8 μm is relatively not important, so, we set the weight as 0.2. As shown in Figure 2(b), when *h*_ZnS_1_ and *h*_ZnS_2_ are 1.336 μm and 1.493 μm, and *h*_IST_ is 11 nm, higher spectral IR emissivity modulation abilities $\Delta {\bar{\varepsilon }}_{3-5}=0.843$ and $\Delta {\bar{\varepsilon }}_{8-14}=0.921$ are obtained in 3–5 μm and 8–14 μm, respectively. It’s interesting to note in Figure 2(c) that controlling the fill factor *f* by laser or electrical excitation enables continuous modulation of the polarization-averaged average emissivity from 0 to ∼0.9 within two IR detection bands. Figure 2(d) indicates the robustness of IR emission capability of the MCTE at different incident angles even up to 70°.

**Figure 3:**
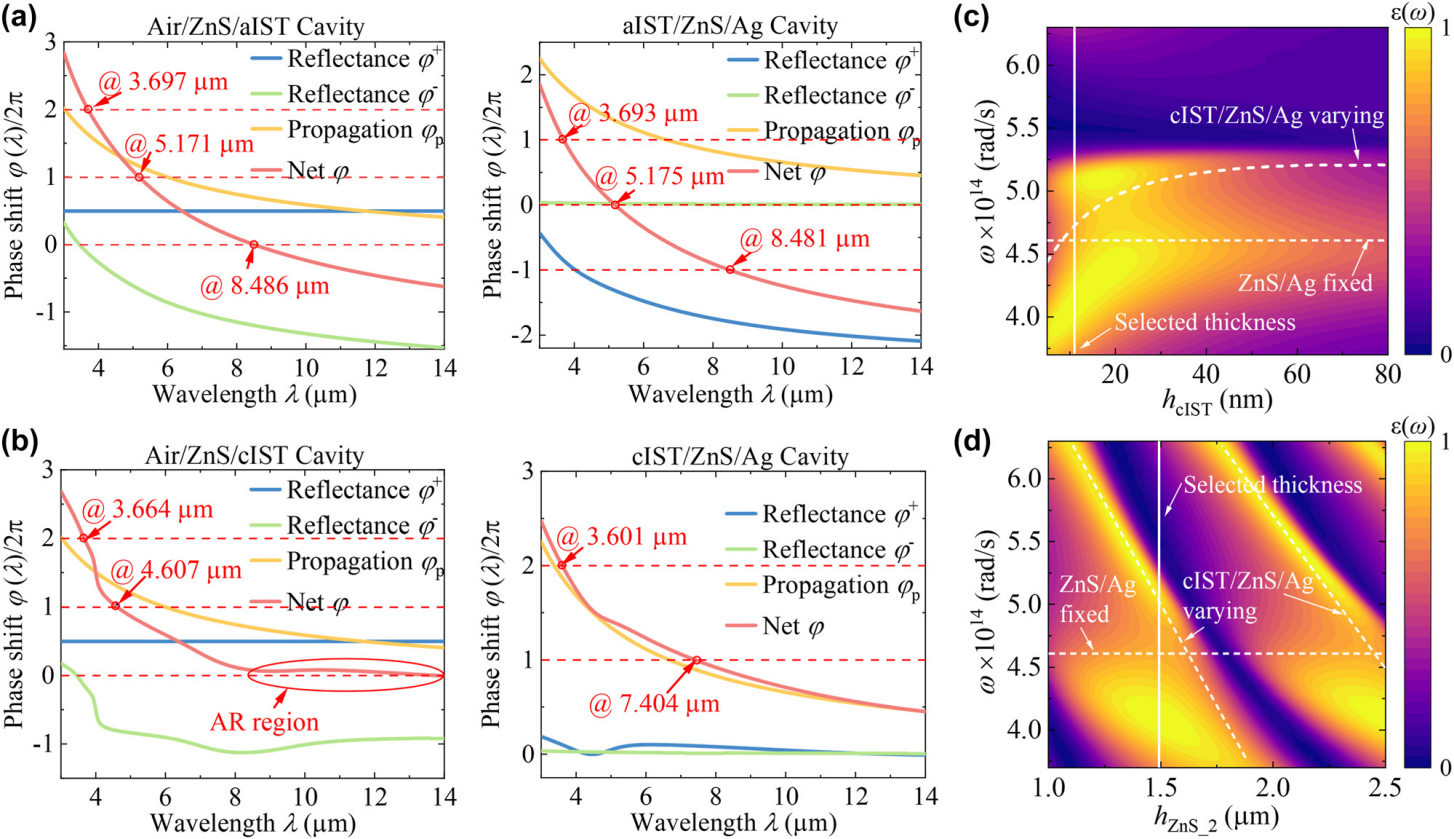
The net phase shift and spectral emissivity as a function of *h*_cIST_ and *h*_ZnS_2_. Spectral phase shifts associated with (a) air/ZnS/aIST and aIST/ZnS/Ag nanocavities, and (b) air/ZnS/cIST and cIST/ZnS/Ag nanocavities; Spectral emissivity as a function of (c) *h*_IST_, and (d) *h*_ZnS_2_, the white line is the selected *h*_cIST_ and *h*_ZnS_2_ thickness and the white short dash lines are the resonance peaks of independent structures.

To further illustrate the mechanism for IR emissivity modulation, we plot the net phase shifts of Air/ZnS/IST and IST/ZnS/Ag nanocavities in Figure 3(a) and (b), which allows us to extract resonances such as the Fabry–Perot (F–P) resonance with net phase equal to a multiple of 2π [42]. Due to the lossless dielectric property of aIST and ZnS, it slightly absorbs energy through Ag substrate, resulting in extremely low emissivity, corresponding to the calculated peak emissivity wavelengths (3.697 μm, 5.184 μm, and 8.447 μm) in Figure 2(b). When the IST layer is crystalline, the net phase shift of top ZnS layer approaches zero at 8–14 μm IR region, meaning that the ZnS layer can be modelled as anti-reflectance (AR) layer. The net phase shift in cIST/ZnS/Ag cavity is 2π at 7.404 μm, revealing that wideband high emission is excited by the resonance coupling within 8–14 μm. The detailed emission enhancement of the top ZnS AR layer is also shown in Supplementary S4. The difference is that the resonances of MCTE at 3.715 μm and 4.609 μm are excited by single mode coupling at 3.601 μm, 3.664 μm and 4.607 μm between upper and lower cavities.

Figure 3(c) clearly shows the process of optical mode coupling from strong to weak coupling (even decoupling) with increasing thickness of cIST layers (*h*_cIST_). The short dash lines represent resonance peaks of independent cIST/ZnS/Ag cavity and AR ZnS layer/Ag as a function of *h*_cIST_. As *h*_cIST_ of the middle cIST layer increases, it becomes more and more difficult for above two modes to penetrate the cIST layer, and thus finally embody the single-peak resonance of AR layer. Figure 3(d) shows the spectral emissivity as function of different *h*_ZnS_2_. A periodic variation of resonances is clearly observed and further indicates that the broadband high emission within 3–5 μm is induced by coupling of multiple FP modes.

### 2.3 VIS-IR compatible colored thermal emitter and optimization

As discussed above, high dual-band IR emission is excited by the resonance mode coupling of top AR layer and middle IST/dielectric/Ag nanocavity. Due to the nature of resonance mode coupling, this enhancement effect does not depend on special materials or complex structures. Therefore, by replacing single ZnS AR layer with a DBR that combines infrared transparent materials ZnS, Ge, and GaAs in a customized design, we prove it is possible to achieve a wide range of colors in VIS spectrum while also ensuring large emissivity variations in infrared detection band at the same time. More importantly, the IR emission regulation mentioned above does not affect the VIS color characteristics.

For the VIS-IR compatible thermal emitter optimization and design, two target fitness value *F*_adopt_ can be expressed as:

(5a) $$F_{\text{adopt\_VIS}}=\min\left(\sqrt{{\left(x-x_{\text{aim}}\right)}^{2}+{\left(y-y_{\text{aim}}\right)}^{2}}\right)$$

(5b) $$\begin{array}{ll} & F_{\text{adopt\_IR}}=\max\left(0.4\left|\Delta {\bar{\varepsilon }}_{3-5}\right|-0.2\left|\Delta {\bar{\varepsilon }}_{5-8}\right|+0.4\left|\Delta {\bar{\varepsilon }}_{8-14}\right|\right) \end{array}$$

where *x*, *y*, *x*_aim_, *y*_aim_ are the CIE 1931 *xy* chromaticity coordinates of colored MCTE’s color and aim color.

**Figure 4:**
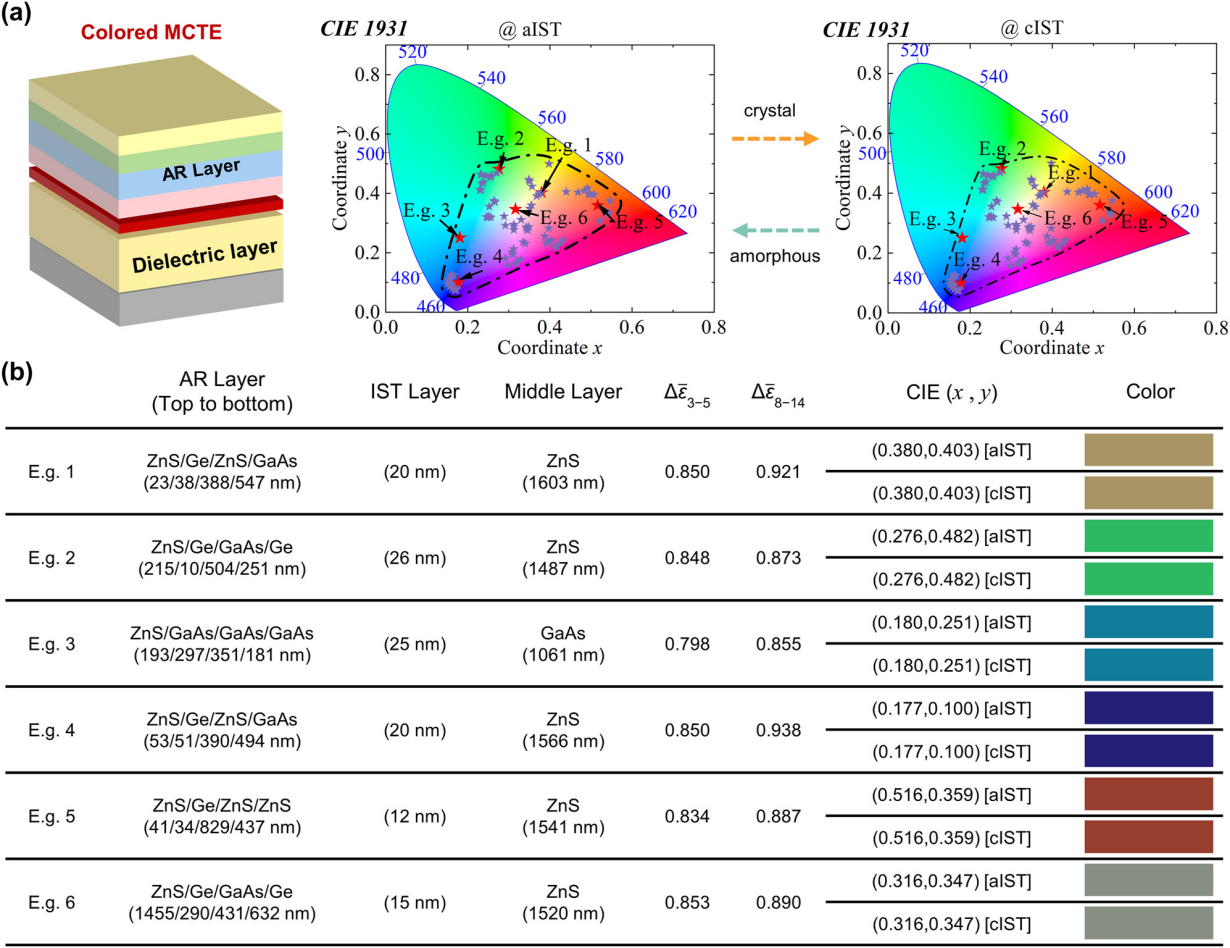
The modulation of color characteristic of colored MCTE. (a) Schematic structure of colored MCTE and its VIS color characteristic range before and after phase-change progress. E.g. 1–E.g. 6 are the six examples with different color characteristics; (b) structures parameters, IR emissivity modulation capability, and color of six examples in (a).

By optimizing the thickness and selected materials of each layer, Figure 4(a) vividly illustrates the enormous potentials of colored MCTE to represent almost all VIS colors, such as earthy yellow, grass green, sea blue, and so on. Because the thickness of IST is sufficiently thin and its imaginary part of permittivity in VIS region is almost constant regardless of the phase of IST, the color of the structure stays unchanged during the phase change (see several examples in Figure 4(a)). Therefore, the interference between the change of VIS color characteristics and IR dual-detection-band emissivity can be avoided. Figure 4(b) shows the corresponding structure parameters, IR dual-band emissivity modulation capability, and color characteristics (see spectral data in Supplementary S5). Our colored MCTE with DBR manifests VIS-IR compatibility, and more importantly shows excellent angular and thickness robustness with large and continuous IR emissivity regulation ability and similar VIS color even at large angles (see more details in Supplementary S6).

### 2.4 Programmable VIS-IR camouflage/anti-counterfeiting performance

Now, we will demonstrate the applications in colored IR thermal camouflage and anti-counterfeiting. Traditional adaptive thermal emitters can only realize a few discrete states, and the scope of regulation is extremely limited, while we can programmable and continuously modulate the IR emissivity of each element by setting different fill factor *f.* Here, we give two examples on how to realize the VIS-IR thermal camouflage and anti-counterfeiting.

**Figure 5:**
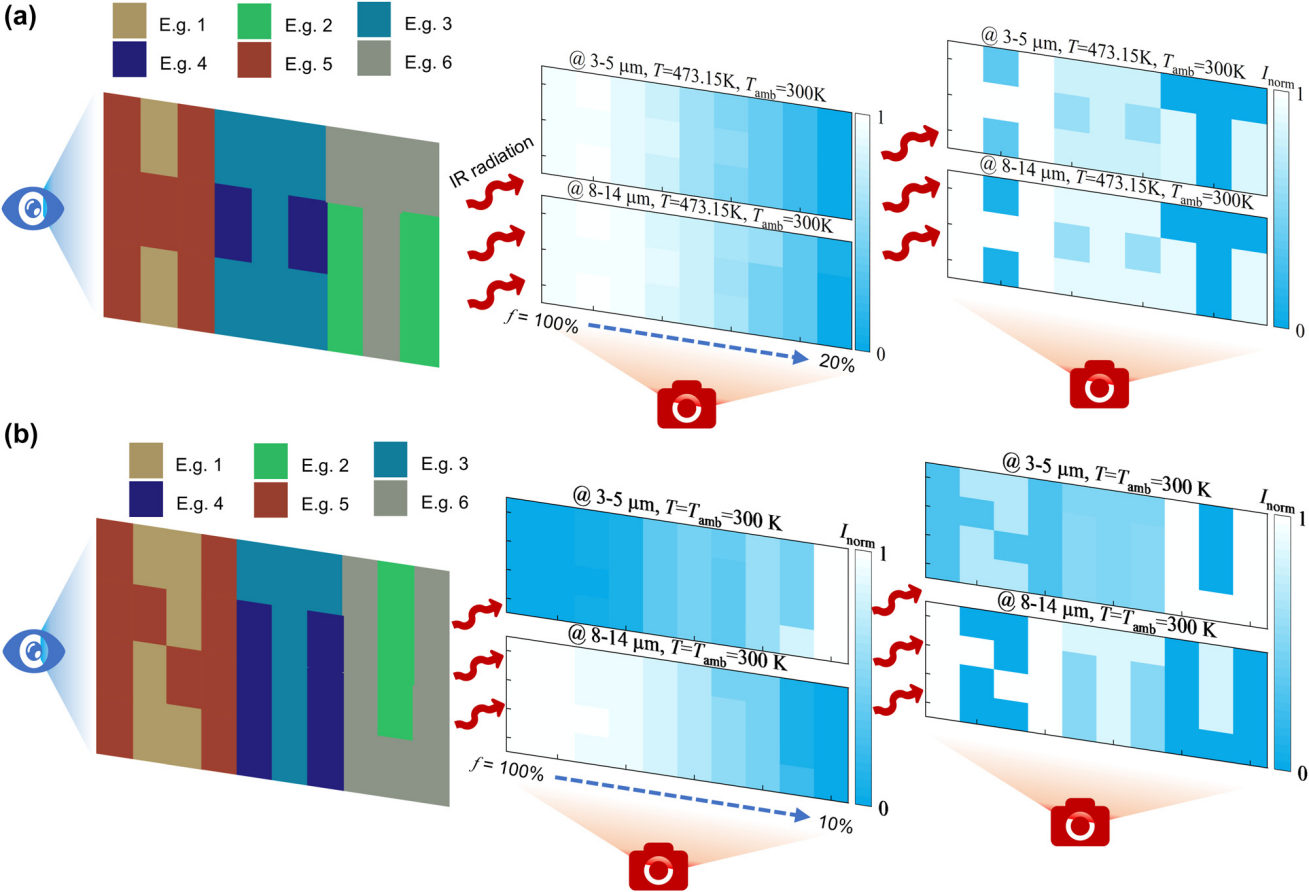
The camouflage and anti-counterfeiting performance. (a) Example of VIS-IR compatible camouflage with colored MCTEs of E.g. 1–6. The left panel is the theoretical VIS image of ‘HIT’, the middle panel is the theoretical IR image as a function of the fill factors *f* from 100 % to 20 % at *T* = 473.15 K and *T*_amb_ = 300 K, and the right panel is the mosaic IR camouflage images when the fill factors *f* of E.g. 1–E.g. 6 are 30 %, 70 %, 70 %, 50 %, 100 %, 20 %, respectively. (b) The example of IR anti-counterfeiting performance with the colored MCTEs of E.g. 1–6. The left panel is the theoretical VIS image of ‘NTU’, the middle panel is the theoretical IR image as a function of the fill factor *f* from 100 % to 20 % at *T* = *T*_amb_ = 473.15 K, and the right panel is the IR images when the fill factors *f* of E.g. 1–E.g. 6 are 30 %, 70 %, 70 %, 50 %, 100 %, 20 %, respectively.

We firstly demonstrate an application example of our designed E.g. 1–E.g. 6 MCTEs in VIS-IR compatible camouflage in Figure 5(a) [43]. Using existing camouflage methods with visual obfuscation capability, we arrange and combine multiple elements of E.g. 1–E.g. 6 MCTEs to construct a 3 × 9 grid image of ‘HIT’. Similarly, we can also arrange them as more complex images as required. For IR programmable and continuous camouflage, the self-radiation plays a dominant role so that each cell with high IR emissivity displays higher radiation intensity when camouflaged object exhibits a higher temperature *T* = 473.15 K which is lower than the phase change temperature of IST (above ∼ 573.15 K). The middle panel of Figure 5(a) clearly shows the trend of IR thermal images of different cells of grid structure with the fill factor changing from 100 % to 20 %. Therein, we can programmable modulate complex IR thermal camouflage images by changing the fill factor of each element to confuse in complex environments, i.e., previously mentioned “thermal mosaic camouflage”. The right panel of Figure 5(a) exhibits the effect of this method that we realize the ‘HIT’ IR thermal image at 3–5 μm and 8–14 μm by setting the fill factor *f* of E.g. 1–6 as 30 %, 70 %, 70 %, 50 %, 100 %, 20 %, respectively. We can also set up to achieve more complex patterns to avoid advanced IR detection to achieve perfect deception.

More importantly, we can also achieve potential infrared anti-counterfeiting performance while achieving thermal mosaic camouflage at a lower temperature. When the camouflaged object exhibits a low temperature *T* = 300 K, the self-radiation at 3–5 μm region (∼5.86 W/m^2^) is much lower than the direct solar radiation, as shown in Figure 1(c). This phenomenon results in the cells with high emissivity showing lower IR thermal radiation intensity which is different with the IR results at 8–14 μm, see middle IR image in Figure 5(b). With the fill factor *f* decreasing from 100 % to 10 %, the IR radiation intensity is enhanced at 3–5 μm while is suppressed at 8–14 μm. As shown in the right IR mosaic camouflage images of Figure 5(b), the word ‘U’ is brightest at 3–5 μm while the word ‘N’ is brightest at 8–14 μm, even if they exhibit identical fill factor at the same location. Compared with the high-temperature structure in Figure 5(a), it should exhibit nearly identical IR images with the same fill factor. This outstanding performance provides a feasible way to realize thermal anti-counterfeiting by using temperature while achieve thermal camouflage by emissivity modulation, which has not been mentioned before.

## 3 Conclusions

In summary, by using the non-volatile phase change and metalloid optical characteristics of IST phase change material within entire IR spectral region, we propose an industry-friendly, lithography-free, and processable multilayer colored MCTE to achieve VIS-IR compatible multi-spectral thermal camouflage and IR anti-counterfeiting. Based on resonance mode coupling, we demonstrate that outstanding IR modulation ability does not depend on a special structure composition so that we can achieve almost all colors based on near 90 % IR emissivity modulation amplitude at 3–5 μm and 8–14 μm by reasonably design the thickness of each layer and the material composition of AR layers. More importantly, the programmable and continuous modulation at IR 3–5 μm and 8–14 μm can be achieved by changing the phase change fill factor *f*, which is the key for realization of IR programmable decoy camouflage or anti-counterfeiting. Our strategy offers a new and convenient platform and method for applications in programmable thermal radiation devices.

## Supplementary Material

Supplementary Material Details
