# Supplementary material for: Colored thermal camouflage and anti-counterfeiting with programmable In3SbTe2 platform
Source: Nanophotonics. 2024 Feb 28;13(6):945–54. doi: 10.1515/nanoph-2023-0924 (PMC11501152; doi:10.1515/nanoph-2023-0924)
Supplement: Supplementary file 1 — Supplementary Material Details [file j_nanoph-2023-0924_suppl_001.docx]

**Colored Thermal Camouflage and Anti-Counterfeiting with Programmable In_3_SbTe_2_ Platform**

Sihong Zhou^1,2,3^, Shikui Dong ^1,2^, Qinghui Pan ^1,2^, Yong Shuai^1,2,*^ and Guangwei Hu^3,*^

^1^Key Laboratory of Aerospace Thermophysics of Ministry of Industry and Information Technology, Harbin 150001, China

^2^School of Energy Science and Engineering, Harbin Institute of Technology, Harbin 150001, China

^3^School of Electrical and Electronic Engineering, 50 Nanyang Avenue, Nanyang Technological University, Singapore, 639798, Singapore

^*^Corresponding author: guangwei.hu@ntu.edu.sg, shuaiyong@hit.edu.cn

**S1. The MODTRAN setting parameters, and the direct solar radiation and self-radiation intensity.**

In our letter, we used the US Standard 1976 atmosphere model, the detailed parameters are shown in Table S1:

Table. S1 MODTRAN’s parameters in our letter

| Parameters |  |
| --- | --- |
| Water Column [cm] | 1762.3 |
| Ozone Column [cm] | 0.34356 |
| CO2 [ppmv] | 400 |
| CO [ppmv] | 0.15 |
| CH_4_ [ppmv] | 1.8 |
| Ground Temperature [K] | 300 |
| Ground Albedo | 0 |
| Aerosol Model | Desert |
| Visibility [km] | 75.75 |
| Sensor Altitude [km] | 1 |
| Sensor Zenith [deg] | 180 |

According to these parameters, we can calculate the direct solar spectral radiation *I*_solar_(*λ*) and the blackbody thermal radiation intensity at 3-5 μm and 8-14 μm shown as following:

**Fig. S1** The solar radiation intensity calculated by MODTRAN and the blackbody radiation intensity at 300K or 473.15K.

When the object temperature is 300K, the self-radiation intensity is lower than the direct solar radiation. Therefore, in the radiation signal received by the detector, the solar radiation occupies the main position, and the device with a higher emissivity will reflect lower solar radiation, so the infrared image displayed by the detector will be shown as a lower signal intensity. On the contrary, when the object temperature is up to 473.15 K, the self-radiation intensity is much higher than the direct solar radiation. So, the higher the emissivity of the object, the stronger the overall radiation signal.

**S2. The GPU-TMM-GA optimization method**

The design and optimization method comprises a GPU parallel TMM for optical characteristic calculation [1], including the emissivity of the 3-5 μm and 8-14 μm infrared detection band and the reflectivity of the 0.36-0.83 μm visible band before and after the phase change, and GA optimization for structure design. By sorting the sample results in each iteration, the samples with better results are selected just as the big yellow ball. Then the parameters of the selected samples are crossover, and the samples of the next iteration are generated until the final result meets the design requirements.

**
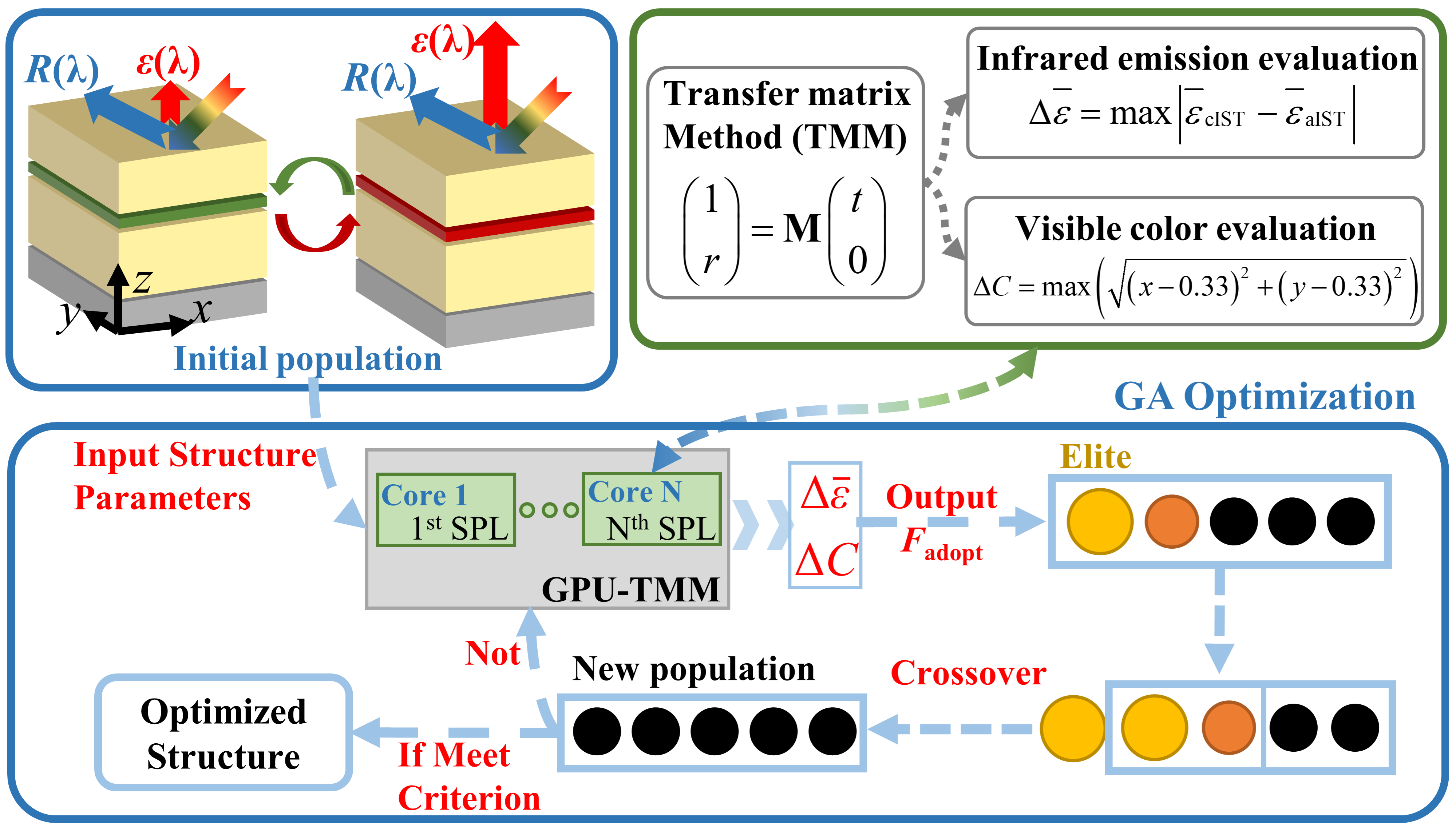
**

**Fig. S2** Process diagram of GPU-TMM-GA optimization method.

[1] Zhou S.H., Guo Y.M., Zhu L.W., et al. Continuous programmable mid-infrared thermal emitter and camouflage based on the phase-change material In3SbTe2. Optics Letters, 2023, 48(16): 4388-4391.

**S3. The relative permittivity.**

The amorphous phase permittivity of IST is considered as a Tauc-Lorentz model and the crystalline phase permittivity of IST is considered as a Tauc-Lorentz-Drude model, which are both from Heßler’s paper at Nature Communications [1]. The detailed Tauc-Lorentz model with the imaginary part of the amorphous IST is described as:

$$\varepsilon_{\text{aIST}\_\text{imag}}=\frac{A}{\omega}\frac{\omega_{0}\gamma\left(\omega-\omega_{g}\right)^{2}}{\left(\omega^{2}-\omega_{0}^{2}\right)^{2}+\omega^{2}\gamma^{2}}\Theta\left(\omega-\omega_{g}\right)$$

where *A* is the resonator strength, *ω* is the angular frequency, *ω*_0_ is the resonance frequency, *γ* is the resonator damping, *ω*_g_ is the band gap frequency, and the Θ(*ω-ω*_g_) is the Heaviside function. And the real part of permittivity of the amorphous IST can be calculated by the Kramers-Kronig relations. While, for the crystalline phase permittivity of IST, a Drude term should be added to describe the metal-like properties of the crystalline phase IST:

$$\begin{matrix} \varepsilon_{\text{cIST\_real}}\text{=real}\left(\frac{\omega_{p}^{2}}{\omega\left(\omega+i\gamma_{D}\right)}\right)+\varepsilon_{\text{aIST\_real}} \\ \varepsilon_{\text{cIST\_imag}}\text{=imag}\left(\frac{\omega_{p}^{2}}{\omega\left(\omega+i\gamma_{D}\right)}\right)+\varepsilon_{\text{aIST\_imag}} \end{matrix}$$

where real(*ω*) means taking the real part of the results, imag(*ω*) means taking the imaginary part of the results, *ω_p_* is the plasma frequency, and *γ_D_* is the Drude damping.

The results of the amorphous phase permittivity of IST and the crystalline phase permittivity of IST are shown in Fig. 1a-b. Besides, the detailed permittivity of ZnS, Ge, GaAs, and Ag are also shown in Figure S1c-f, which are obtained from Ref [2-5].


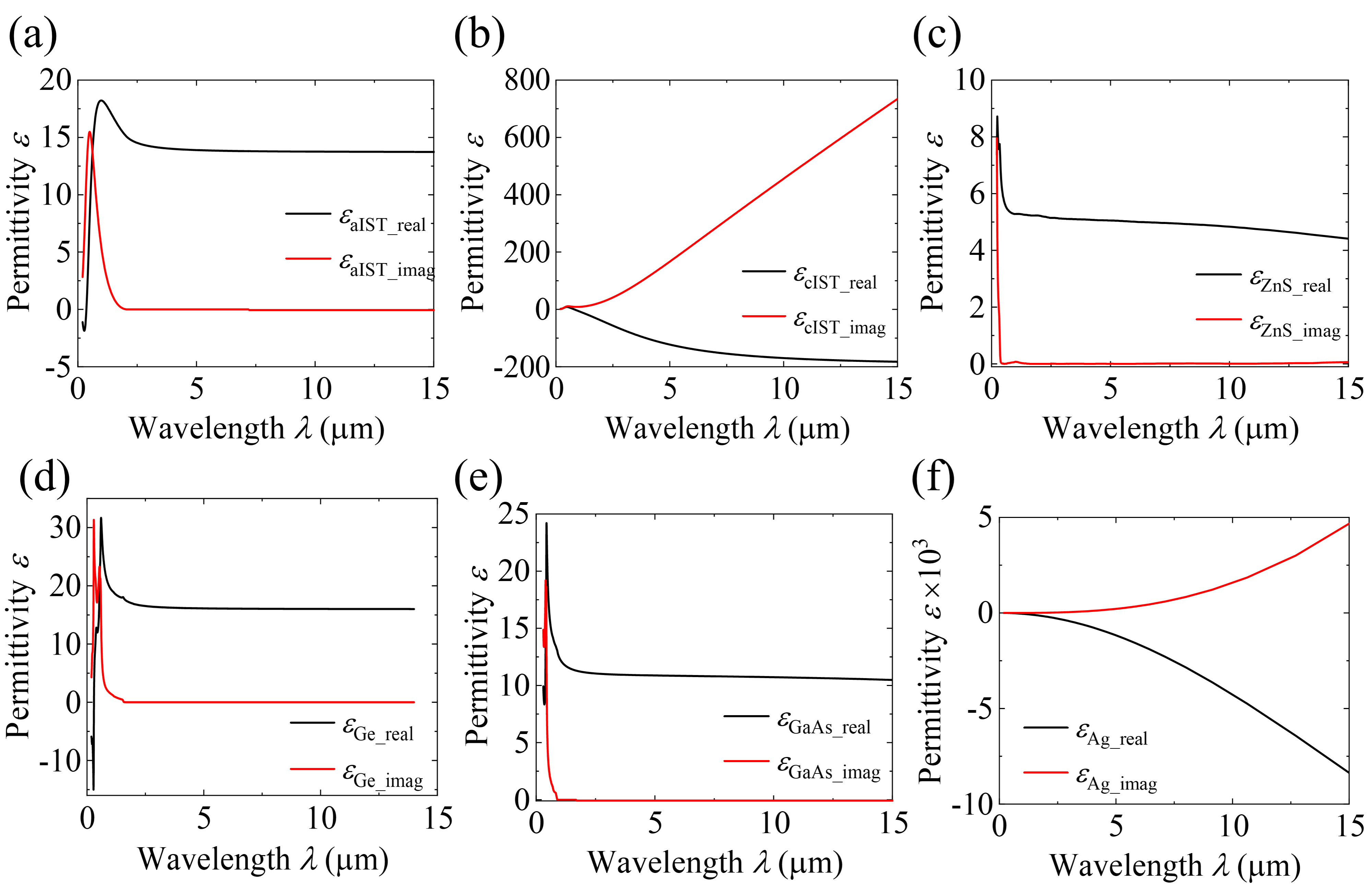


**Fig. S3.** The permittivity of aIST [1], cIST [1], ZnS [2], Ge [3,4], GaAs [5], and Ag [6].

[1] Heßler, A.; Wahl, S.; Leuteritz, T., et al. In_3_SbTe_2_ as a programmable nanophotonics material platform for the infrared. Nature Communications 2021, 12 (1), 1-10.

[2] Querry M. R. Optical constants of minerals and other materials from the millimeter to the ultraviolet. Contractor Report CRDEC-CR-88009, 1987.

[3] Nunley T. N., Fernando N. S., Samarasingha N., et al. Optical constants of germanium and thermally grown germanium dioxide from 0.5 to 6.6 eV via a multi-sample ellipsometry investigation. Journal of Vacuum Science and Technology B 2016, 34, 061205.

[4] Burnett J. H., Kaplan S. G., Stover E. Refractive index measurements of Ge. Proc. SPIE 9974, Infrared Sensors, Devices, and Applications VI, 99740X, 2016.

[5] Palik E. D. Handbook of Optical Constants of Solids. Academic Press, 3, 1998.

[6] Ciesielski A., Skowronski M., Trzinski M., et al. Controlling the optical parameters of self-assembled silver films with wetting layers and annealing. Applied Surface Science 2017, 421 (B), 349-356.

**S4. The more detailed information for the enhancement of IR emission**

Fig. S4(a) and (b) shows the normalized electric field intensity at these resonance wavelengths, indicating the out-of-phase (odd) and inphase (even) superposition of the resonant modes corresponding to the antisymmetric and symmetric modes at strong coupling regime.

At the IR 8-14 μm region, the top ZnS layer acts as an AR layer and reduces the reflectance. Fig. S4(c) clearly demonstrates the emissivity of our MCTE with/without top ZnS AR layer.


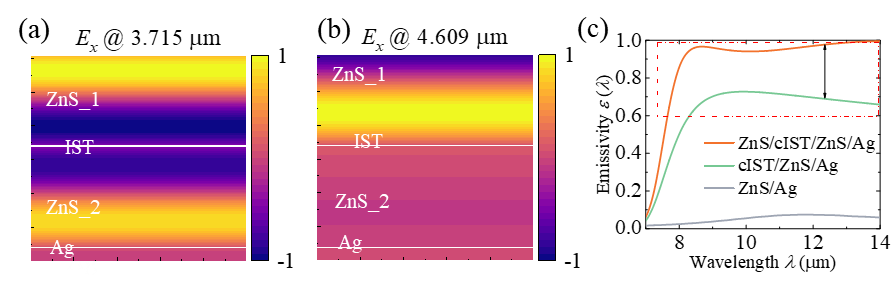


**Fig S4.** The electric filed intensity *E*_x_ at two resonance wavelengths (a) 3.715 μm and (b) 4.609 μm. (c) The spectral emissivity of MCTE with/without top ZnS layer and the spectral emissivity of ZnS AR layer/Ag.

**S5. The visible color and IR emissivity of E.g. 1-6**

Based on the resonance mode coupling, we can achieve almost all color characteristics under the premise that the infrared dual detection band still has a high emission control ability by reasonably replacing different anti-reflection layers. In the manuscript, we take six examples for verification. The detailed visible reflectance results at 0.36-0.83 μm and the infrared emissivity results at 3-14 μm of E.g.1-6 are shown in Fig. S4. It is clear demonstrated from the results that all six samples can achieve a regulatory potential of close to 0.9 in the detection bands of 3-5 μm and 8-14 μm, while, displaying different visible colors.

**
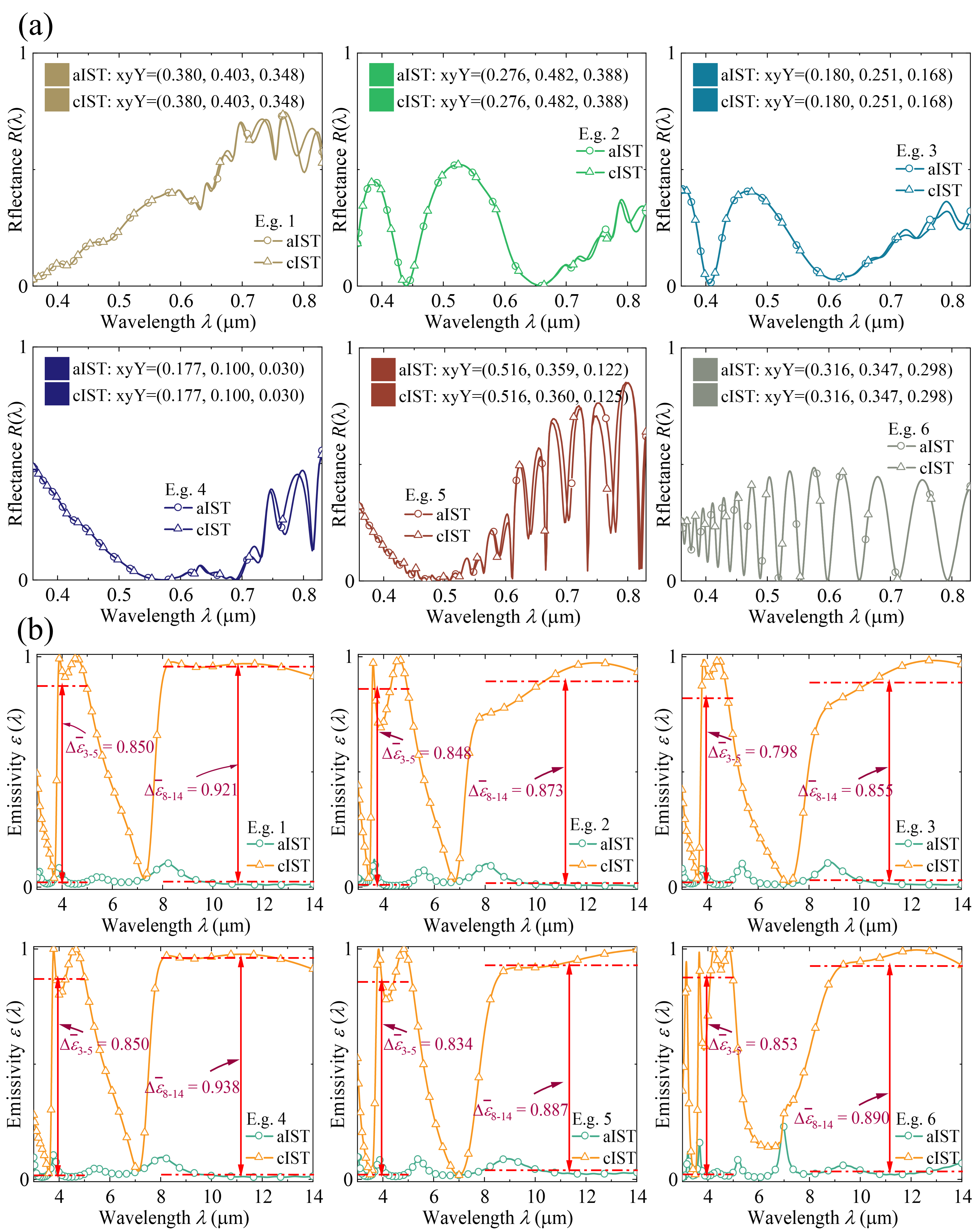
**

**Fig. S5** (a) The spectral reflectance of E.g. 1-E.g. 6. (b) The spectral emissivity of E.g.1-E.g.6 before and after phase change.

**S6. The visible and infrared optical characteristics of E.g. 1**

The structure schematic of E.g. 1 is shown in Fig. S6a (a) which is consisted with a ZnS/Ge/ZnS/GaAs AR layer above IST/ZnS/Ag nanocavity. Fig. S6a (b) exhibits the robustness of the color of the E.g. 1 at a large incident angle. With the incident angle increasing, the color will gradually become lighter, but it will still be yellow.

The polarization-averaged average emissivity of E.g. 1 can still be continuously adjusted effectively by adjusting the fill factor *f* of the local phase transition region. Fig. S6a (c) vividly shows the continuously modulation emissivity at 3-5 μm and 8-14 μm, which is similarly with the original ZnS/IST/ZnS/Ag MCTE. And the IR spectral emissivity also can be maintained at a large incident angle, as shown in Fig. S6a (d).


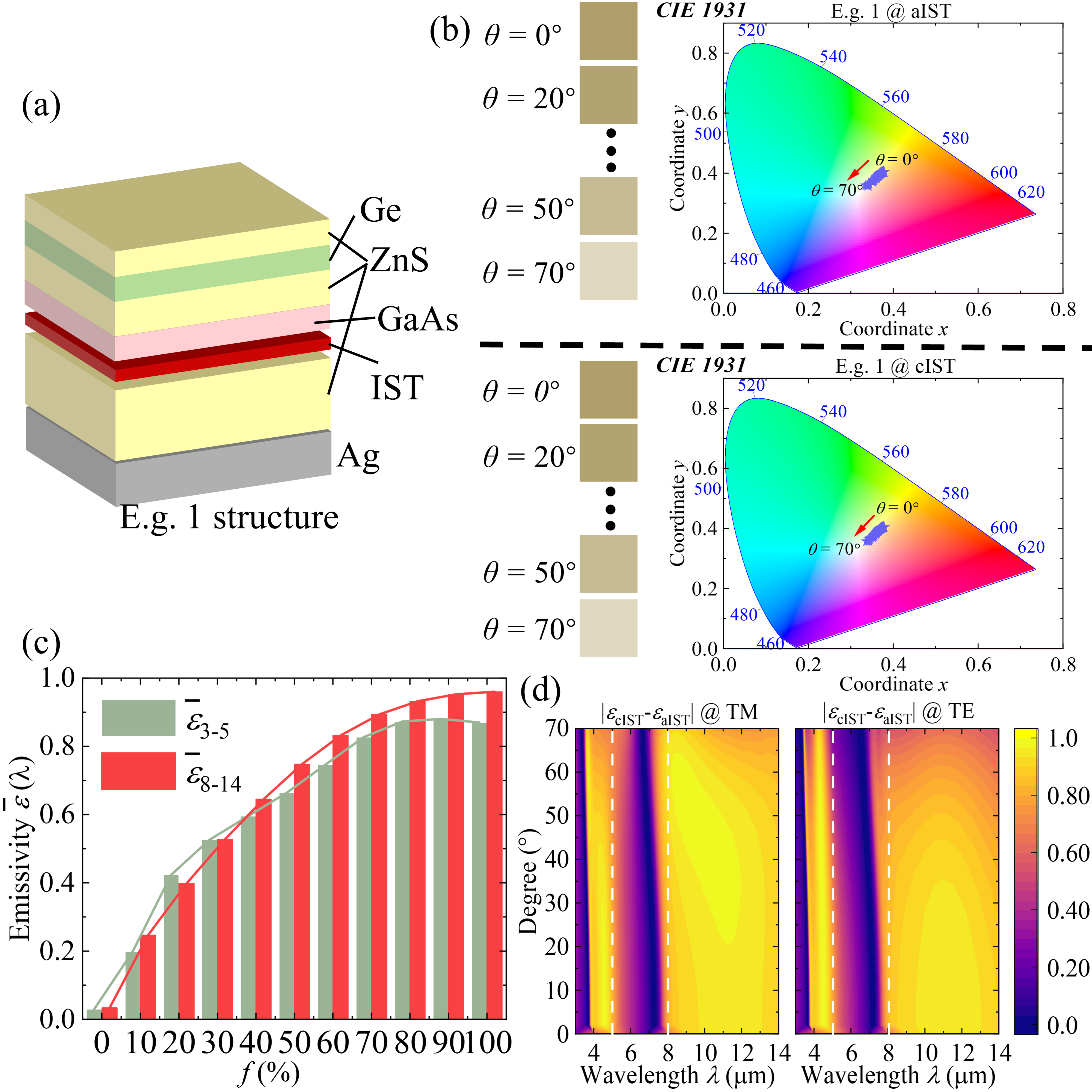


**Fig. S6a** (a) The structure schematic of E.g. 1. (b) The visible color of E.g. 1 under different incident angles. (c) the polarization-averaged average emissivity of E.g. 1 with different fill factor *f* at 3-5 μm and 8-14 μm. (d) The spectral emissivity difference of E.g. 1 at different polarization under different incident angles.

The robustness of thickness of each layer is also discussed below. Fig. S6b (a) and (c) show the mean and standard deviation results of spectral emissivity and reflectance of E.g. 1 MCTE by increasing and decreasing the thickness of each layer by 5%. Fig. S6b (b) and (d) more clearly shows the results of infrared emissivity modulation and reflect color characteristics. When the thickness of each layer increases and decreases by 5%, the infrared emissivity modulation ability changes no more than 4.36% in 3-5 μm and 2.97% in 8-14 μm and they can show the nearly same colors.

**
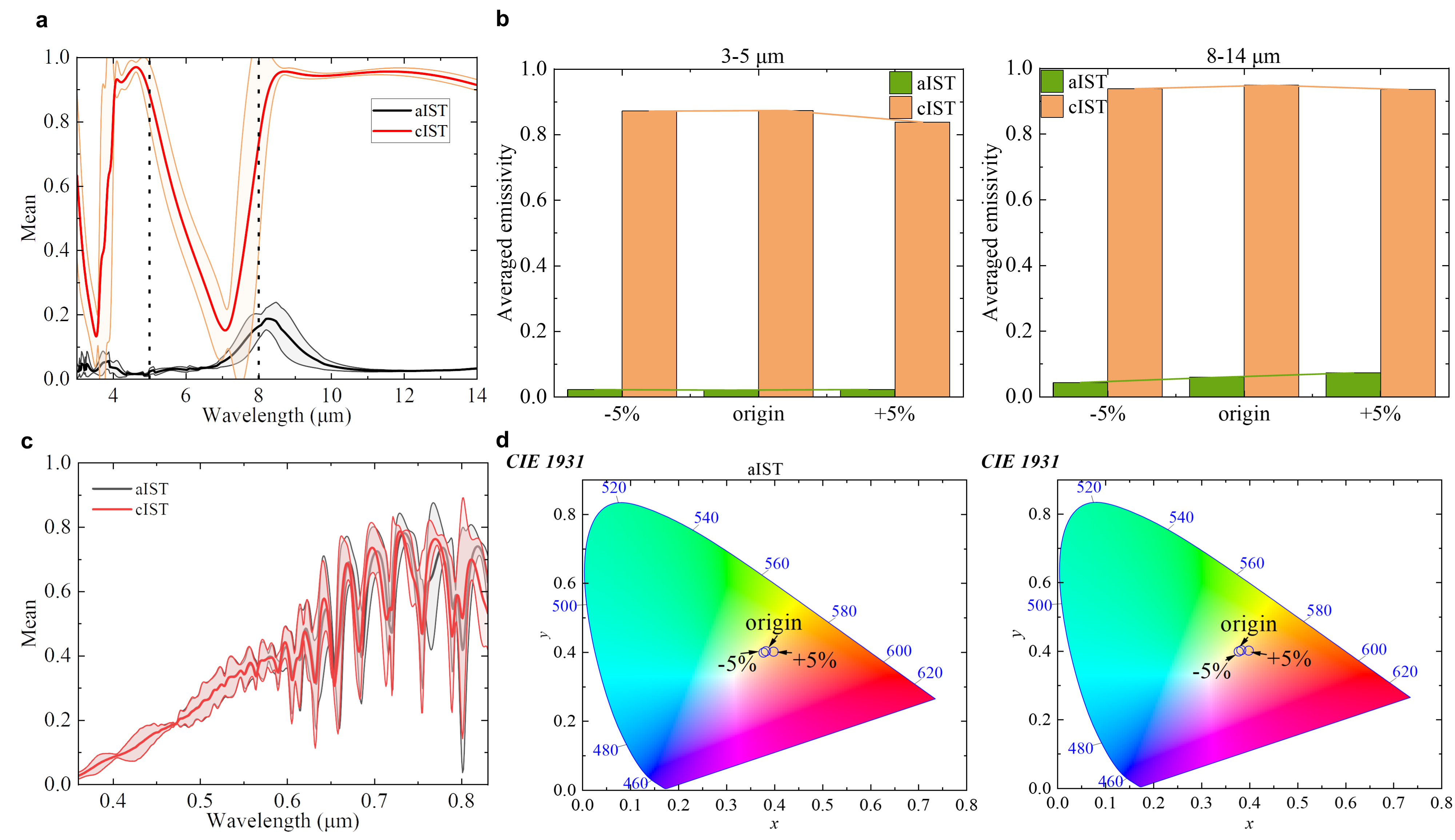
**

**Fig. S6b (**a). The mean and standard deviation results of spectral emissivity of E.g. 1 MCTE by increasing and decreasing the thickness of each layer by 5%; (b). Averaged emissivity of E.g. 1 MCTE by increasing and decreasing the thickness of each layer by 5%; (c). The mean and standard deviation results of spectral reflectance of E.g. 1 MCTE by increasing and decreasing the thickness of each layer by 5%; (d). The color characteristics of E.g. 1 MCTE by increasing and decreasing the thickness of each layer by 5%.
